# The Role of 20% Ethanol in Enhancing Pterygium Surgery Outcomes: A Clinical Study

**DOI:** 10.7759/cureus.65830

**Published:** 2024-07-31

**Authors:** Rekha R Mudhol, Shilpa K

**Affiliations:** 1 Ophthalmology, Shri BM Patil Medical College, Bijapur Lingayat District Education (BLDE) (Deemed to be University), Vijayapura, IND

**Keywords:** pterygium excision, corneal astigmatism, conjunctival autograft, 20% ethanol, pterygium

## Abstract

Introduction

Pterygium is an ocular surface disorder characterized by a hyperplastic growth of conjunctiva encroaching over the cornea. It causes redness, watering, and foreign body sensation. Surgical excision is the preferred mode of treatment when there is encroachment over the visual axis, chronic irritation, restricted ocular motility, and cosmetic concerns. Various surgical methods have been adopted for the treatment and to prevent recurrences. This study assessed the effectiveness and safety of 20% ethanol as an adjuvant in pterygium excision with conjunctival autograft implantation, evaluating surgical outcomes.

Methods

A prospective hospital-based interventional study was conducted among 30 patients with pterygium from August 2022 to December 2023. Patients were evaluated preoperatively for anterior segment, posterior segment, visual acuity, and corneal astigmatism. Pterygium was excised using 20% ethanol as an adjuvant, and conjunctival autograft was placed over the bare sclera without sutures. Patients were evaluated on postoperative days 1, 8, 30, and 90 for graft condition, visual acuity, corneal astigmatism, and associated complications.

Results

After three months of follow-up, the mean visual acuity improved to LogMAR 0.46±0.35 (p=0.001), which was statistically significant, and the average corneal astigmatism decreased from 3.36±2.87 to 0.87±0.57 (p=0.0001). No recurrence was noted among the participants.

Conclusion

This study has shown that using 20% ethanol as an adjuvant for pterygium excision facilitated clean dissection of a pterygium from the underlying cornea and the pterygium-induced corneal astigmatism has significantly decreased, which led to progress in vision.

## Introduction

Pterygium is a common ocular surface disorder, which was described by Duke-Elder as a triangular-shaped degenerative and hyperplastic process characterized by encroachment of the bulbar conjunctiva over the cornea, which can occur on the medial and lateral side in the palpebral aperture [1]. It is more commonly seen nasally than temporally. Pterygium is caused by excessive exposure to ultraviolet (UV) radiation in sunlight. Dry and dusty environments also contribute to the development of pterygium.

The prevalence of pterygium in India was 13.2% [2], and the prevalence is elevated in the "pterygium belt", which spans from 30 degrees north to 30 degrees south of the equator [3].

Pterygia can be asymptomatic or cause redness, watering on exposure to heat, foreign body sensation, or disturbance in vision. It causes induced astigmatism by flattening the central cornea through traction caused by the pterygium. This leads to the development with the rule of astigmatism. There is also a pooling of tear films between the pterygium apex and cornea, which can induce astigmatism [4].

Surgical excision is the preferred management for pterygium. The indications for pterygium excision include encroachment over the visual axis, chronic irritation, restricted ocular motility, and cosmetic concerns. Various surgical techniques have been developed over the years, which include bare sclera excision, conjunctival autograft implantation, limbal cell transplantation, and amniotic membrane transplantation. Adjuvants like beta irradiation, thiotepa, intra- or postoperative mitomycin C, cyclosporine, 5-fluorouracil, and anti-vascular endothelial growth factor (VEGF) have been used over the years to get better surgical outcomes [5,6]. The recurrence of pterygium is one of the complications observed after its surgical excision. Pterygium excision using the bare sclera technique has a higher recurrence rate of 24-89% [7]. The rate of recurrence has decreased with the use of different types of adjuvants. Using mitomycin C through various routes reduced the recurrence rate to 2-16% [8]. However, the application of mitomycin C has sight-threatening complications like scleral melt and inflammatory scleritis [9].

In ophthalmology, dilute alcohol is commonly employed to remove the epithelium in photorefractive keratectomy (PRK) and laser subepithelial keratomileusis (LASEK). The application of dilute ethanol delaminates the corneal epithelium and leaves behind a very smooth surface, which helps in the epithelization of the cornea [10]. In tissues, it causes apoptosis of cells and protein denaturation. Proteins or peptides like cytokines, enzymes, and growth factors can undergo rapid denaturation by ethanol. It disrupts the basement membrane and damages hemidesmosome junctions between corneal epithelium [11]. This study assesses the effectiveness, safety, and surgical outcomes of using 20% ethanol as an adjuvant in pterygium excision with conjunctival autograft.

## Materials and methods

A prospective, hospital-based interventional study evaluated the efficacy, safety, and surgical outcomes of intraoperative application of 20% ethanol as an adjuvant in pterygium excision with conjunctival autograft. This study was conducted among the 30 eyes of 30 patients with primary pterygium whose pterygium grade was I or more than I and aged more than 18 years who had come to ophthalmology OPD, Bijapur Lingayat District Education (BLDE)'s Shri BM Patil Medical College Hospital and Research Centre, Vijayapura. This study was carried out from August 2022 to December 2023. Patients with recurrent pterygium, pseudopterygium, prior ocular surgery, meibomitis, atopic keratoconjunctivitis, Sjogren's syndrome or herpetic keratitis, other ocular infections, and any retinal pathologies were included in the exclusion criteria. Patients who were unwilling to follow up were also excluded from the study. Patients with cataracts did not undergo any cataract surgery during the study period. Institutional ethical clearance and the patient's willful consent were taken.

Information regarding the patient's age, sex, occupation, symptoms, and visual acuity, laterality of the disease, the position of the pterygium (nasal, temporal, or both), the keratometry values of horizontal and vertical meridian, and astigmatism were collected. Snellen's chart was used to assess visual acuity and then converted to LogMAR. The auto refractometer measured the vertical and horizontal meridian. Corneal astigmatism was measured by calculating the difference between vertical and horizontal meridians. The anterior segment was examined by a slit lamp biomicroscope.

Pterygium was graded according to the extent of corneal involvement as grade I (pterygium crossing the limbus), grade II (pterygium head is at the midway between the limbus and pupillary margin), grade III (pterygium at the pupillary margin), and grade IV (pterygium crossing the pupillary margin) [4].

Surgical technique

**Figure 1:**
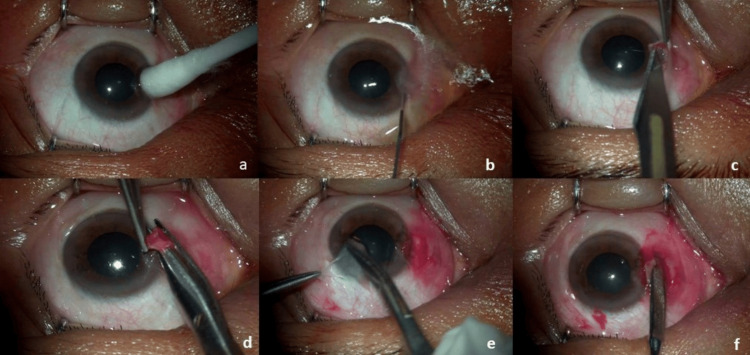
Steps in pterygium excision using 20% ethanol as an adjuvant with conjunctival autograft: (a) application of 20% ethanol over the pterygium head for 60 seconds, (b) it is thoroughly washed with normal saline, (c) the head is dissected using a 15-number blade, (d) pterygium is excised, (e) graft is taken from the superior temporal conjunctiva, and (f) graft is placed over the bare sclera and pressure is applied over five minutes

Surgery was performed under peribulbar anesthesia by a single surgeon. A cotton bud was dipped in a container containing 20% ethanol and applied for 60 seconds over the pterygium head. The ocular surface was washed thoroughly with normal saline. The pterygium head is lifted using forceps and dissected using a scalpel with a 15-number blade. The time taken for the pterygium head dissection was noted. The remaining pterygium tissue was dissected from the underlying sclera and excised. The graft was harvested from the superior temporal conjunctiva of the same eye. The dimensions of the graft were measured using a caliper, which was taken 1 mm beyond the size of the bare sclera. Bleeding was allowed, and a graft was positioned over the bare sclera. The pressure was applied over the graft using forceps for five minutes or more to ensure that the graft was adherent to the bare sclera. Two drops of moxifloxacin eye drops were put in just before patching (Figure 1). In the case of double-headed pterygium, using a caliper, the required graft size was measured from both the temporal and the nasal sides of the bare sclera after pterygium excision. The graft dimensions for both sides were marked adjacently on the superior temporal conjunctiva, ensuring it was 1 mm larger than the bare sclera. The graft was then dissected and split into two halves. One half was placed over the bare sclera on the nasal side, and the other half was applied to the temporal side, maintaining limbus to limbus orientation.

The initial examination was done on the first postoperative day, with subsequent follow-up visits done on the postoperative days 8, 30, and 90. The following parameters were assessed during follow-up visits: graft condition, ocular symptoms, visual acuity, corneal astigmatism, and complications.

Data analysis

This data was analyzed using IBM SPSS Statistics for Windows, V. 20.0 (IBM Corp., Armonk, NY). The results are displayed using mean±SD, percentages, and graphs. Repeated measures of the Friedman test were used to compare pre- and postoperative variables. The statistical significance was considered when the p-value was less than 0.05. All statistical tests were performed in two-tailed.

## Results

The study involved 30 eyes from 30 patients, with a mean age of 47.06±13.20 years. The majority of the participants were within the age group of 50-59 (eight patients, 26.7%). Most were females (22 patients, 73.3%), while eight were males (26.7%). Fifteen (50%) patients had a primary pterygium in their right eye, and 15 (50%) patients had it in their left eye. Pterygium is graded into four grades depending on the extent of pterygium over the cornea using the slit lamp examination. There were two (6.7%) patients with grade I pterygium, 21 (70%) with grade II pterygium, six (20%) with grade III pterygium, and one (3.3%) patient with grade IV pterygium. Double-headed pterygium was present in three (10%) patients; one (3.3%) patient had temporal pterygium, and the rest of the patients (86.67%) had nasal pterygium (Table 1).

**Table 1:** Demographic characteristics of patients enrolled in pterygium surgery

Demographic characteristics	No. of patients
Age (in years)	
Mean+SD	47.06+13.20
Range	26-72
Gender	
Males	8 (26.7%)
Females	22 (73.3%)
Laterality of eye	
Right eye	15 (50%)
Left eye	15 (50%)
Grade of pterygium	
Grade I	2 (6.7%)
Grade II	21 (70%)
Grade III	6 (20%)
Grade IV	1 (3.3%)
Type of pterygium	
Nasal pterygium	26 (86.67%)
Temporal pterygium	1 (3.3%)
Double-headed pterygium	3 (10%)

**Figure 2:**
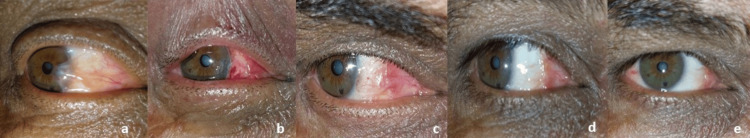
Preoperative and postoperative follow-up of a patient with grade II nasal pterygium: (a) patient with grade II nasal pterygium, (b) postoperative day 1, (c) postoperative day 8, (d) postoperative day 30, and (e) postoperative day 90

All the patients underwent pterygium excision using 20% ethanol as an adjuvant with conjunctival autograft. The dissection of the pterygium from the underlying cornea was easy and clean, with an average dissection time of 82.83±39.78 seconds. In cases where the graft was thicker due to the presence of tenon's tissue, a longer duration of the application of pressure was required to ensure the graft was adherent to the bare sclera. The average duration of pressure application over the graft was 321.33±55.07 seconds. No complications were noted intraoperatively. Patients were followed up on postoperative days 1, 8, 30, and 90 (Figure 2). Patients were instructed to use carboxymethyl cellulose 0.5% eye drops and a combination of gatifloxacin 0.3% and prednisolone 1% eye drops four times daily for three months following surgery.

**Figure 3:**
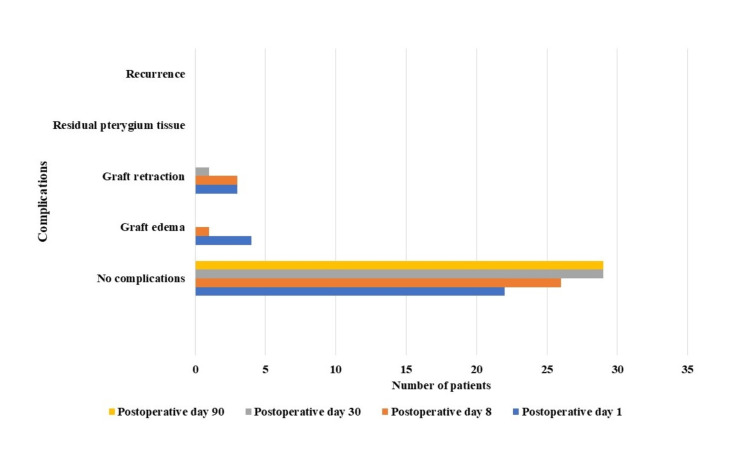
Complications following pterygium excision with conjunctival autograft on postoperative days 1, 8, 30, and 90

On postoperative day 1, all patients experienced watering, photophobia, and pain, which subsided by day 8. Among the 30 patients, 23 (76.6%) patients had graft well adherent to the sclera with no complications, four (13.3%) patients had graft oedema, and three (10.0%) patients had graft retractions. On postoperative day 8, 26 (86.7%) patients had graft well adherent to the sclera with no complications, one (3.3%) patient had graft oedema, and three (10.0%) patients had graft retraction. During one month of follow-up, one (3.3%) patient had graft retraction. In this study, patients with graft retraction had one or two of the graft borders (nasal and inferior or superior) retracted which were noticed on initial follow-up periods. On subsequent follow-up periods, adjacent conjunctiva might have grown and integrated with the graft which showed a reduced number of patients with graft retraction. Patients had no signs of recurrence or other complications at three months of follow-up (Table 2, Figure 3).

**Table 2:** Complications following pterygium excision using 20% ethanol as an adjuvant with conjunctival autograft implantation on different postoperative days

Complications	Day 1	Day 8	Day 30	Day 90
No complications	23 (76.6%)	26 (86.7%)	29 (96.7%)	30 (100%)
Graft oedema	4 (13.3%)	1 (3.3%)	0	0
Graft retraction	3 (10.0%)	3 (10.0%)	1 (3.3%)	0
Residual pterygium tissue	0	0	0	0
Recurrence	0	0	0	0

**Figure 4:**
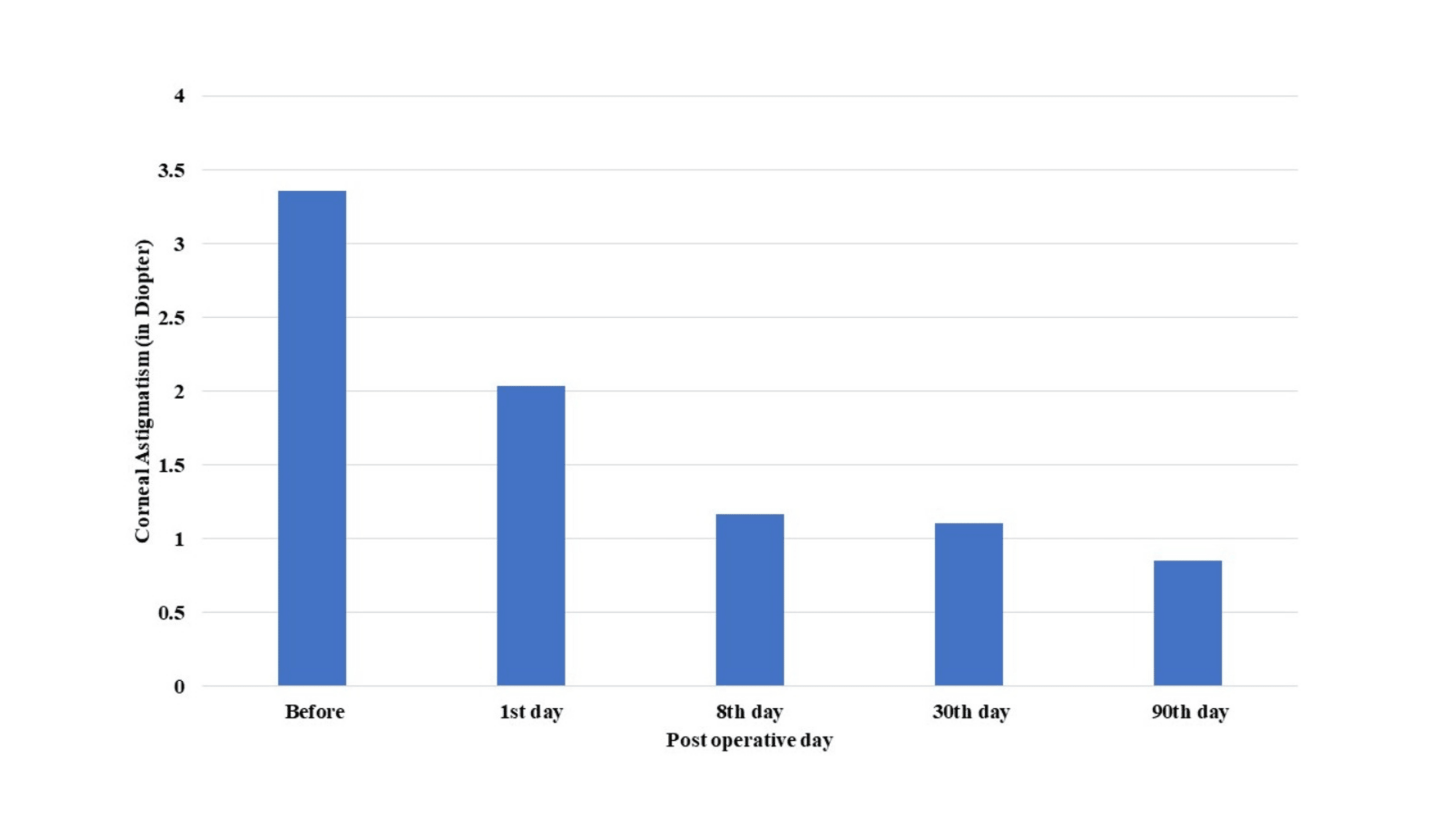
Graph showing corneal astigmatism preoperatively and postoperative days 1, 8, 30 and 90

**Figure 5:**
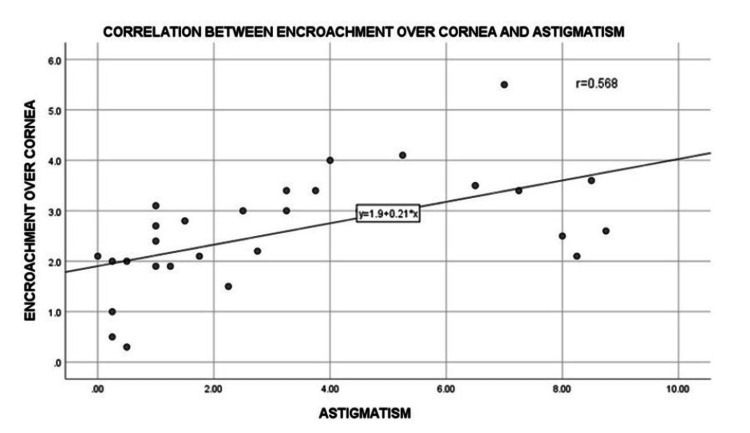
Graph showing the correlation between the encroachment over the cornea and corneal astigmatism preoperatively

There was a statistically significant improvement in the mean visual acuity from 0.72±0.47 LogMAR preoperatively to 0.45±0.35 LogMAR in the third month of follow-up post-surgery (p=0.001) (Table 3). Additionally, there was an increase in the K1 value (vertical meridian) from 42.43±2.83 D before surgery to 44.12±1.35 D at three-month follow-up post-surgery, which was statistically significant (p=0.008) (Table 4). There was an increase in the K2 value (horizontal meridian) from 45.72±1.88 D on preoperative day to 46.00±2.35 D on postoperative day 1. This might be attributed to postoperative inflammation following surgery which led to increased tear production (watering), causing an increase in the horizontal keratometry value on postoperative day 1. There were a decrease in the K2 value (horizontal meridian) on postoperative day 8 (45.23±1.57 D), no noticed change on postoperative day 30 (45.23±1.58 D), and a further decrease in the K2 value to 43.65±7.55 D on postoperative day 90 (p=0.001) (Table 5). Corneal astigmatism showed a statistically significant reduction from 3.36±2.87 D on preoperative day to 2.03±1.36 D, 1.17±1.02 D, 1.11±1.02 D, and 0.85±0.57 D on postoperative days 1, 8, 30, and 90, respectively (p=0.0001) (Table 6, Figure 4). Furthermore, there was a moderately positive correlation between encroachment of the pterygium over the cornea and corneal astigmatism before the surgery (Pearson correlation coefficient, r=0.568) (Figure 5).

**Table 3:** Visual acuity in LogMAR before and after pterygium excision using 20% ethanol as an adjuvant with conjunctival autograft

	Visual acuity (LogMAR)	Friedman test	Significant value
				Preoperative	0.72±0.47	25.35	0.001
Postoperative day 1	0.39±0.34						
Postoperative day 8	0.48±0.37						
Postoperative day 30	0.43±0.35						
Postoperative day 90	0.45±0.35						

**Table 4:** Vertical meridian (K1) in diopters before and after pterygium excision using 20% ethanol as an adjuvant with conjunctival autograft

	K1 value	Friedman test	Significant value
				Preoperative	42.43±2.83	13.898	0.008
Postoperative day 1	44.04±1.56						
Postoperative day 8	44.09±1.39						
Postoperative day 30	44.12±1.32						
Postoperative day 90	44.12±1.35						

**Table 5:** Horizontal meridian (K2) in diopters before and after pterygium excision using 20% ethanol as an adjuvant with conjunctival autograft

	K2 value	Friedman test	Significant value
				Preoperative	45.72±1.88	35.347	0.001
Postoperative day 1	46.00±2.35						
Postoperative day 8	45.23±1.57						
Postoperative day 30	45.23±1.58						
Postoperative day 90	43.65±7.55						

**Table 6:** Corneal astigmatism in diopters before and after pterygium excision using 20% ethanol as an adjuvant with conjunctival autograft

	Corneal astigmatism	Friedman test	Significant value
				Preoperative	3.36±2.87	37.804	0.0001
Postoperative day 1	2.03±1.36						
Postoperative day 8	1.17±1.02						
Postoperative day 30	1.11±1.02						
Postoperative day 90	0.85±0.57						

## Discussion

UV exposure is one of the primary risk factors for the development of pterygium. Consequently, it is highly prevalent in Karnataka due to high levels of UV exposure and the high prevalence of outdoor occupations among the population. Various surgical methods are employed for pterygium excision and to prevent recurrences. Pterygium excision with conjunctival autograft and several adjuvants have been used to reduce recurrences. In this study, 20% ethanol was used as an adjunctive for pterygium excision.

Alcohol-assisted delamination has been used in ophthalmology for conditions such as recurrent corneal erosion, refractive surgery (laser-assisted in situ keratomileusis (LASIK)), corneal collagen cross-linking, and epithelial/anterior stromal dystrophies [12]. Ethanol delaminates the corneal epithelial cells and creates a smooth and clean dissection plane between the epithelium and Bowman's layer [12]. Few studies were conducted to study the role of ethanol in pterygium surgery. Chen and Hsu used an optical zone marker with a barrel filled with two drops of 20% ethanol, which was adsorbed using a cellulose sponge after 60 seconds. After the pterygium excision, ethanol was applied over the bare sclera. Chen and Hsu observed a recurrence rate of 5.3% with no other intraoperative or postoperative complications, whereas mitomycin C had a recurrence rate of 10.0% [11]. Tsumi et al. placed a metal ring well over the pterygium head, and 2-3 drops of 20% ethanol were applied inside the ring for 40 seconds. They concluded that it was a simple and safe procedure to create a plane that helps to separate the pterygium and the cornea [13]. Nowacka and Lubinski performed sliding flap surgery with adjunctive 0.05% mitomycin C or 20% ethanol, noting a 13% recurrence rate in the alcohol group with no other complications due to alcohol [14]. Wu et al. noted that 20% ethanol made removing the residual pterygium tissue easier after blunt dissection of the pterygium head. During follow-up periods of three months, only one case had a recurrence with no intraoperative and postoperative complications, and two cases had a recurrence in the control group [15]. In this study, the application of 20% ethanol helps in the easy and clean dissection of the pterygium from the underlying cornea. Strong adhesions present over the cornea were easily dissected. There were no residual tissues of pterygium over the cornea. Corneal epithelium healed when patients came for a one-week follow-up. There were no intraoperative and postoperative complications with no signs of recurrence.

Pterygium causes a decrease in visual acuity due to the obstruction in the visual axis, such as in grade IV pterygium, and by the induced corneal astigmatism. In this study, the mean preoperative corneal astigmatism was 3.36±2.87 D, which declined to 2.03±1.36 D on postoperative day 1 and further to 0.85±0.57 D on postoperative day 90, with this reduction being statistically significant (p=0.0001). There were also a decrease in the K2 value and an increase in the K1 value after surgery, suggesting that removing pterygium, which induced flattening of the cornea, allowed the cornea to return to its normal shape.

Garg et al. reported a reduction in astigmatism from 3.47±1.74 D to 1.10±0.78 D 90 days following surgery, noting that pterygium excision surgery using conjunctival graft and amniotic membrane had a better reduction in astigmatism compared to the bare sclera technique [16]. In contrast, Nowacka and Lubinski observed that the reduction of corneal astigmatism was statistically insignificant (p>0.05) in the sliding flap technique using 20% ethanol [14]. Mohammad-Salih and Sharif concluded that the length and area of the pterygium had a closer association with corneal astigmatism compared to the width of the pterygium, observing that a 2 D of corneal astigmatism was seen in a pterygium patient when the length is more than 2.2 mm, width more than 5 mm, or total area more than 6.25 mm^2^ [17]. This study also found a moderately positive correlation between the length of the pterygium and corneal astigmatism. The patient with grade III pterygium and 4.1 mm encroachment over the cornea had an astigmatism of 5.25 D, while the patient with 2.1 mm encroachment had an astigmatism of 1.25 D in this study.

A reduction in corneal astigmatism contributed to improving visual acuity. Misra et al. observed the preoperative best-corrected visual acuity improved from 6/7.25 to 6/6 following pterygium excision using conjunctival autograft, along with a decrease in topographic astigmatism [18]. Similarly, this study also showed a significant improvement in vision from preoperative day LogMAR 0.72±0.47 to LogMAR 0.45±0.35 after three months of follow-up.

This study demonstrated a good surgical outcome, characterized by improved vision and reduced corneal astigmatism, with no recurrence and good intraoperative and postoperative outcomes. Most recurrences tend to develop within three months following surgery. However, recurrences can still occur for up to one year, which might be less than the early postoperative period. This study’s short follow-up period and smaller sample size might make it difficult to draw a long-term conclusion on the pterygium recurrences and other surgical outcomes. A longer follow-up period and a larger sample size could provide more comprehensive insights into the recurrences of pterygium and the long-term outcome of the surgery, thereby reducing potential biases and yielding more generalized findings.

## Conclusions

Using 20% ethanol as an adjuvant has shown to be a safe and cost-effective technique of pterygium excision compared to other adjuvants such as mitomycin C and anti-VEGF bevacizumab. It helps in the easy dissection of the pterygium head from the underlying cornea. There were good surgical outcomes associated with this technique. In this study, there were no signs of recurrence or intraoperative or postoperative complications. There was a significant decline in corneal astigmatism induced by pterygium, thus improving visual acuity. Thus, 20% ethanol can be used as a cost-effective alternative technique in pterygium excision.

## References

[1] Hill JC, Maske R (1989). Pathogenesis of pterygium. Eye (Lond).

[2] Tandon R, Vashist P, Gupta N (2022). The association of sun exposure, ultraviolet radiation effects and other risk factors for pterygium (the SURE RISK for pterygium study) in geographically diverse adult (≥40 years) rural populations of India -3rd report of the ICMR-EYE SEE study group. PLoS One.

[3] Detels R, Dhir SP (1967). Pterygium: a geographical study. Arch Ophthalmol.

[4] Maheshwari S (2003). Effect of pterygium excision on pterygium induced astigmatism. Indian J Ophthalmol.

[5] Alpay A, Uğurbaş SH, Erdoğan B (2009). Comparing techniques for pterygium surgery. Clin Ophthalmol.

[6] Janson BJ, Sikder S (2014). Surgical management of pterygium. Ocul Surf.

[7] Youngson RM (1972). Recurrence of pterygium after excision. Br J Ophthalmol.

[8] Shahraki T, Arabi A, Feizi S (2021). Pterygium: an update on pathophysiology, clinical features, and management. Ther Adv Ophthalmol.

[9] Rubinfeld RS, Pfister RR, Stein RM (1992). Serious complications of topical mitomycin-C after pterygium surgery. Ophthalmology.

[10] Oh JY, Yu JM, Ko JH (2013). Analysis of ethanol effects on corneal epithelium. Invest Ophthalmol Vis Sci.

[11] Chen KH, Hsu WM (2006). Intraoperative ethanol treatment as an adjuvant therapy of pterygium excision. Int J Biomed Sci.

[12] Dua HS, Deshmukh R, Ting DS, Wilde C, Nubile M, Mastropasqua L, Said DG (2021). Topical use of alcohol in ophthalmology - diagnostic and therapeutic indications. Ocul Surf.

[13] Tsumi E, Levy J, Pitchkhadze A, Baidousi A, Lifshitz T (2012). New approach for pterygium removal using 20 % ethanol. Int Ophthalmol.

[14] Nowacka B, Lubiński W (2019). Recurrence rate and corneal astigmatism after ‘sliding flap’ technique with intraoperative application of 0.05% mitomycin C or 20% ethanol for pterygium surgery. Klin Oczna/Acta Ophthalmol Polonica.

[15] Wu XN, Jiang L, Tang NN (2021). 20% ethanol assists the excision of primary pterygium. Int Eye Sci.

[16] Garg P, Sahai A, Shamshad MA, Tyagi L, Singhal Y, Gupta S (2019). A comparative study of preoperative and postoperative changes in corneal astigmatism after pterygium excision by different techniques. Indian J Ophthalmol.

[17] Mohammad-Salih PA, Sharif AF (2008). Analysis of pterygium size and induced corneal astigmatism. Cornea.

[18] Misra S, Craig JP, McGhee CN, Patel DV (2014). A prospective study of pterygium excision and conjunctival autograft with human fibrin tissue adhesive: effects on vision, refraction, and corneal topography. Asia Pac J Ophthalmol (Phila).

